# Effects of Stabilization Exercises and Pelvic Floor Muscle Training on Urinary Parameters in Individuals with Chronic Low Back Pain and Urinary Incontinence: A Randomized Controlled Trial

**DOI:** 10.3390/jcm15062333

**Published:** 2026-03-18

**Authors:** İbrahim Küçükcan, Yavuz Yakut

**Affiliations:** 1 Department of Physiotherapy and Rehabilitation, Private Physiotherapy Clinic, Gaziantep 27000, Türkiye; 2 Department of Physiotherapy and Rehabilitation, Faculty of Health Sciences, Hasan Kalyoncu University, Gaziantep 27000, Türkiye; yavuz.yakut@hku.edu.tr

**Keywords:** urinary incontinence, chronic low back pain, stabilization exercises, pelvic floor muscle training

## Abstract

**Background/Objectives**: This randomized controlled trial aimed to investigate the effects of stabilization exercises combined with pelvic floor muscle training (PFMT) on urinary parameters in individuals with chronic low back pain (CLBP) and urinary incontinence. **Methods**: A total of 44 women aged 18–65 years were randomized into three groups: PFMT combined with stabilization exercises, standard PFMT group, and a control group. The intervention groups participated in an 8-week exercise program. Urinary symptoms, anxiety, and quality of life were assessed using validated questionnaires. **Results**: The primary outcome (UDI) demonstrated significantly greater improvement in the PFMT combined with stabilization group compared with both the standard PFMT and control groups (*p* < 0.01). Post-treatment comparisons indicated that both intervention groups showed significant improvements in urinary symptoms and quality of life compared with the control group (*p* < 0.05). **Conclusions**: PFMT combined with stabilization exercises may be an effective approach for improving urinary parameters. Further studies are warranted to better elucidate the efficacy of PFMT combined with stabilization exercises. Trial Registration: ClinicalTrials.gov Identifier: NCT05666427.

## 1. Introduction

Pelvic floor muscle training (PFMT) is a widely used physiotherapy and rehabilitation approach for treating urinary incontinence and has the highest level of evidence among conservative interventions [1,2]. In this approach, various exercises are taught and implemented in a regular regimen to improve the endurance, strength, and coordination of the pelvic floor muscles [3]. Combined exercise programs are also implemented for women with urinary incontinence. In these programs, exercises targeting the abdominal muscles are incorporated along with PFMT. Combined programs are reported to be more effective than standard PFMT [4]. These combined programs, which train the pelvic floor and abdominal regions simultaneously, have been preferred in individuals presenting with both urinary incontinence and chronic low back pain (CLBP) [5].

Previous studies have suggested that CLBP may be associated not only with musculoskeletal dysfunction but also with various pelvic floor–related disorders, including urinary incontinence [5]. Several biomechanical and neuromuscular mechanisms have been proposed to explain this relationship, such as impaired trunk motor control, altered intra-abdominal pressure regulation, and dysfunctional synergy between the pelvic floor muscles and deep abdominal stabilizers [6,7]. Evidence indicates that individuals with CLBP often exhibit delayed or insufficient activation of the pelvic floor and trunk muscles, which may compromise lumbopelvic stability and continence mechanisms simultaneously [8]. These findings support the rationale for therapeutic approaches that target both trunk stabilization and pelvic floor muscle function in this population.

In recent years, epidemiological and clinical studies have increasingly reported a higher prevalence of urinary incontinence among individuals with chronic low back pain compared with the general population, suggesting a complex and potentially bidirectional relationship between these conditions [9]. In addition, deficits in trunk motor control and altered activation patterns of the deep stabilizing muscles have been consistently demonstrated in individuals with CLBP, which may adversely affect both spinal stability and pelvic floor function [10,11]. These findings further emphasize the need for integrative rehabilitation strategies that simultaneously address lumbopelvic stabilization and pelvic floor muscle function in this population.

Although most previous studies addressing urinary problems coexisting with CLBP focus on incontinence, other related conditions, such as prolapse, overactive bladder syndrome, fecal incontinence, irritable bowel syndrome, sexual dysfunction, and leg pain, may also be present and should not be overlooked in scientific research and clinical studies [12].

Various neurochemical, physiological, kinesiological, biomechanical, and functional associations between the urinary system and CLBP have been proposed in previous research, with particular emphasis on the role of the core stabilizing muscles and the pelvic floor muscles in mediating these interactions [13]. Pelvic floor muscle training (PFMT) is a structured, exercise-based physiotherapy intervention designed to improve the strength, endurance, coordination, and timing of pelvic floor muscle contractions. It is widely accepted as a first-line conservative treatment for urinary incontinence [14]. Beyond its local effects on continence, PFMT has been shown to function synergistically with the deep abdominal and trunk stabilizing muscles, contributing to lumbopelvic stability and postural control [3,8]. Evidence suggests that individuals with CLBP often exhibit delayed or insufficient activation of the pelvic floor and trunk muscles, which may simultaneously compromise spinal stability and continence mechanisms [5]. Based on this theoretical and clinical framework, it is hypothesized that a combined intervention incorporating PFMT and trunk stabilization exercises would result in greater improvements in urinary symptoms, quality of life, and related psychosocial outcomes compared with PFMT alone or no intervention.

The objective of this study was to compare the effects of pelvic floor muscle training combined with stabilization exercises, pelvic floor muscle training alone, and no intervention on urinary symptoms, quality of life, and related psychosocial parameters in individuals with chronic low back pain and urinary incontinence.

## 2. Materials and Methods

### 2.1. Study Design and Ethical Considerations

**Figure 1:**
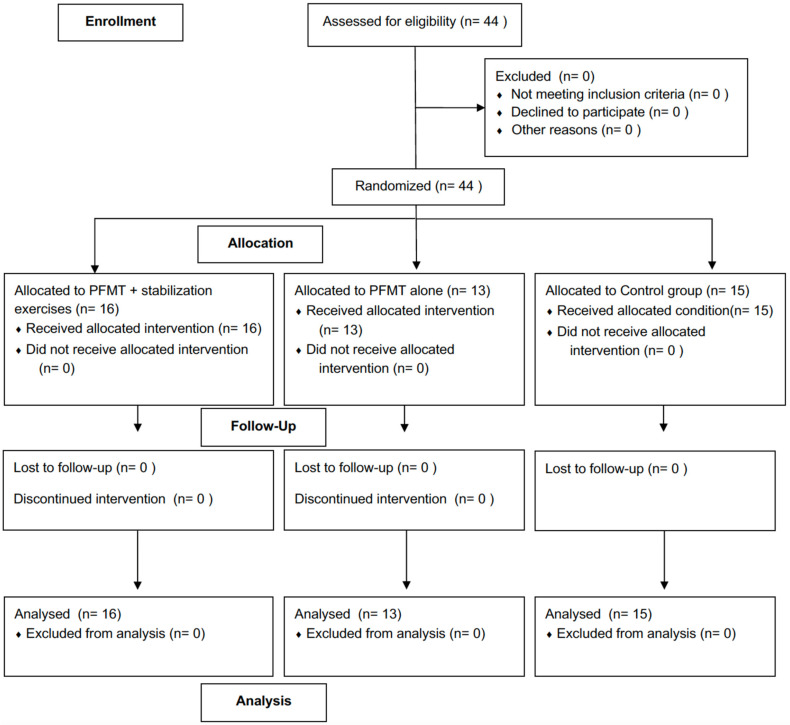
CONSORT flow diagram of participant recruitment, allocation, follow-up, and analysis.

- This study was designed as a randomized controlled trial (RCT). The study protocol was approved by the Hasan Kalyoncu University Faculty of Health Sciences Non-Interventional Research Ethics Committee (Approval No: 2022/005, Date: 10 January 2022). All procedures were conducted in accordance with the principles of the Declaration of Helsinki. The trial was prospectively registered at ClinicalTrials.gov (Registration No: NCT05666427).
- Randomization was performed using a computer-generated random number sequence prepared by an independent researcher not involved in recruitment or assessment. Group assignments were placed in sequentially numbered, opaque, sealed envelopes prepared in advance, kept in a locked cabinet, and opened sequentially after baseline assessment to ensure allocation concealment.
- The study was conducted at a private physiotherapy clinic in Gaziantep, Türkiye. Data collection was conducted during the study period in 2023.
- The study was conducted and reported in accordance with the CONSORT guidelines. A CONSORT flow diagram illustrating participant recruitment, allocation, follow-up, and analysis is provided in Figure 1 [15].

### 2.2. Participants and Inclusion Criteria

Participants were eligible for inclusion if they met the following criteria:

- Presence of urinary incontinence, defined by affirmative answers to the symptom-based questions: “Do you leak urine when you cough, sneeze, or laugh?” and “Do you leak urine with a sudden urge before you can reach the toilet?” [16,17].
- Women aged 18–65 years [18].
- CLBP lasting for at least 3 months, with participants reporting CLBP at least once per week [19].
- A Numeric Rating Scale (NRS) score ≥ 4 [20]. Pain intensity assessed using the NRS was used as an inclusion criterion to ensure that participants had clinically relevant chronic low back pain, which constituted the defining characteristic of the study population.
- An Oswestry Disability Index score ≥ 20% [21].

### 2.3. Exclusion Criteria

- Individuals with radiculopathy [22,23].
- A history of lumbar or pelvic surgery within the past 6 months [21].
- Individuals who had received PFMT within the past 3 months [1].
- Patients with systemic, inflammatory, rheumatic, malignant, bony pathologies, or other conditions that could cause secondary CLBP.
- Individuals with stage 3 or 4 pelvic organ prolapse diagnosed by a physician [17].
- Pregnant women and those who had delivered within the previous 12 months [24].
- Body mass index (BMI) ≥ 30 kg/m^2^ [25].

### 2.4. Trial Registration

This randomized controlled trial was registered at ClinicalTrials.gov (Identifier: NCT05666427) on 29 December 2022.

### 2.5. Ethics Approval

Ethical approval for this study was obtained from the Hasan Kalyoncu University Faculty of Health Sciences Non-Interventional Research Ethics Committee (Approval No: 2022/005, Date: 10 January 2022). All participants provided written informed consent prior to participation.

### 2.6. Evaluation Methods

All outcome assessments were performed at baseline and after completion of the intervention period by the same physiotherapist using standardized protocols.

Urinary symptoms were evaluated using the Urogenital Distress Inventory (UDI) [26], which assesses the severity of urinary symptom-related distress. Participants completed the questionnaire in a quiet clinical setting under the supervision of the assessor, and standardized instructions were provided prior to administration.

Quality of life related to urinary symptoms was evaluated using the Incontinence Quality-of-Life Questionnaire (I-QOL) [27]. Participants were instructed to respond according to their experiences over the previous four weeks, in line with the original questionnaire guidelines.

Incontinence severity was assessed using the Incontinence Severity Index (ISI) [28], a brief and validated measure based on the frequency and amount of urine leakage.

Anxiety levels were assessed using the Trait Anxiety Inventory (TAI), derived from the State-Trait Anxiety Inventory (STAI) [29].

All questionnaires were administered in their validated Turkish versions, and scoring procedures were performed according to the developers’ guidelines.

### 2.7. Intervention Protocol

Randomization was performed using a simple random allocation method. Group assignments were placed in sequentially numbered, opaque, sealed envelopes, which were opened after participant enrollment to ensure allocation concealment, in accordance with standard randomized trial methodology [15]. Participants were randomized into three groups using the sealed-envelope method: a stabilization exercise combined with PFMT group, a standard PFMT group, and a control group. In the stabilization exercises combined with PFMT group, participants received combined pelvic floor muscle training and stabilization exercises for 8 weeks, consisting of one supervised session per week with a physiotherapist and two home-based sessions per week. During this protocol, participants were instructed to perform stabilization exercises in coordination with breathing while simultaneously engaging the pelvic floor muscles [8] (Table 1). The standard PFMT group received conventional pelvic floor muscle training alone. In this protocol, separate contractions were taught for the slow-twitch (type I) and fast-twitch (type II) muscle fibers of the pelvic floor. To improve endurance, activation of the slow-twitch fibers was emphasized, and participants were instructed to visualize the pelvic floor as an elevator: lift up, hold at the top for a period, and then slowly lower. To increase strength, activation of the fast-twitch fibers was emphasized, and participants were instructed to imagine quickly turning a running tap off and on. This training approach is consistent with established PFMT principles targeting fiber-specific adaptations [1]. Both intervention groups followed the same schedule of one supervised session and two home-based sessions per week for 8 weeks [13] (Table 2). In both groups, the program began with exercises tolerated by each participant, with repetitions, sets, contraction hold times, exercise types, and positions progressed from basic to advanced at defined intervals, following established principles of progressive overload and neuromuscular adaptation [30]. Warm-up exercises were performed at the beginning of each session, and cool-down exercises were performed at the end. Rest intervals of 10–30 s were allowed between exercises and 1–3 min between sets. Prior to the exercises, participants were provided with the necessary information using visual presentations and anatomical models. No intervention was provided to the control group. Adherence to the home-based exercise sessions was monitored through weekly verbal confirmation during supervised visits.

Representative examples of the stabilization exercises are provided in the Supplementary Materials (Figures S1 and S2).

### 2.8. Statistical Analysis

Data were analyzed using SPSS 22.0 statistical software [31]. The Kolmogorov–Smirnov test was used to assess normality. As the data were not normally distributed, non-parametric tests were applied. Between-group comparisons were performed using the Kruskal–Wallis test, followed by Mann–Whitney U tests for pairwise comparisons when appropriate. Within-group pre–post comparisons were conducted using the Wilcoxon signed-rank test. A significance level of *p* < 0.05 was adopted for all analyses.

Given the three-group pre–post design, change scores (Δ = post-treatment minus pretreatment) were calculated to further account for baseline differences. Between-group comparisons of change scores were conducted using the Kruskal–Wallis test followed by Mann–Whitney U tests for pairwise analyses when the overall test was significant.

The UDI was considered the primary clinical outcome of this study, consistent with the study objective, which focused on urinary parameters. A power analysis was performed using G*Power (version 3.1.9.7) for a repeated-measures ANOVA (within–between interaction), assuming an effect size f = 0.25, an alpha level of 0.05, power (1 − β) of 0.80, three groups, and two measurement points. The analysis indicated that a minimum total sample size of 42 participants was required. The final sample of 44 participants met the required sample size and achieved an actual power of 0.80. Accordingly, analyses involving UDI scores correspond directly to the predefined primary study hypothesis.

## 3. Results

All randomized participants completed the study. Sixteen participants in the PFMT combined with stabilization exercises group, thirteen in the standard PFMT group, and fifteen in the control group completed the intervention, with no losses or exclusions during the study period.

No significant differences were observed between the groups in age, BMI, or symptom duration, indicating that the groups were homogeneously distributed across these variables. All participants included in the study were female. Detailed demographic and physical characteristics of the participants are presented in Table 3.

### Analysis of Urinary Parameters and Quality of Life

Post-treatment measurements showed significant differences between the groups for all these parameters (*p* < 0.05) (Table 4). In addition to statistical significance, the observed between-group differences reflected clinically meaningful effects, particularly for UDI and I-QOL scores.

Comparisons between the stabilization exercises combined with PFMT group and the standard PFMT group revealed a moderate effect size for UDI (r = 0.46). A small effect size was observed for ISI (r = 0.27). Small effect sizes were also identified for TAI (r = 0.18) and I-QOL (r = 0.15). In the comparison between the stabilization exercises combined with PFMT group and the control group, large effect sizes were observed across multiple urinary and quality-of-life outcomes, including UDI (r = 0.65), ISI (r = 0.73), TAI (r = 0.51), and I-QOL (r = 0.63). In the comparison between the standard PFMT group and the control group, moderate to large effect sizes were observed for UDI (r = 0.37), ISI (r = 0.47), and I-QOL (r = 0.56). In contrast, a small-to-moderate effect size was found for TAI (r = 0.29) (Table 5).

Effect sizes were expressed as r (|z|/√N) with 95% confidence intervals calculated using Fisher’s z transformation. For comparisons between the stabilization exercises combined with PFMT group and the standard PFMT group, the 95% confidence intervals were: UDI (0.10–0.72), ISI (−0.10–0.57), TAI (−0.23–0.50), and I-QOL (−0.23–0.50). For the comparison between the stabilization exercises combined with PFMT group and the control group, the 95% confidence intervals were as follows: UDI (0.39–0.81), ISI (0.51–0.86), TAI (0.19–0.73), and I-QOL (0.36–0.80). For the comparison between the standard PFMT group and the control group, the 95% confidence intervals were: UDI (0.02–0.65), ISI (0.10–0.70), TAI (−0.07–0.58), and I-QOL (0.21–0.74) (Table 5).

To address the baseline imbalance observed in UDI scores, additional analyses were performed using change scores (post-treatment minus pretreatment). Between-group comparisons of ΔUDI demonstrated a statistically significant difference among the three groups (*p* = 0.002), with the stabilization exercises combined with PFMT group showing greater improvement compared to both the standard PFMT and control groups. Similar patterns were observed for ΔISI (*p* < 0.001) and ΔI-QOL (*p* < 0.001), whereas ΔTAI showed a smaller but statistically significant between-group difference (*p* = 0.042). Although post-treatment comparisons between the standard PFMT and control groups reached statistical significance for UDI, change-score analyses indicated that the magnitude of improvement did not differ significantly between the two groups. These findings support the robustness of the post-treatment comparisons and indicate that the results were not solely attributable to baseline differences (Table 6).

No statistically significant differences were observed among the three groups in pretreatment UDI, ISI, TAI, and I-QOL scores, as assessed by the overall Kruskal–Wallis test (*p* > 0.05). However, pairwise comparison revealed a significant baseline difference in UDI scores between the stabilization exercises combined with PFMT group and the standard PFMT group (*p* < 0.05) (Table 7).

When UDI scores were compared between groups, all pairwise comparisons showed significant differences, indicating a reduction in urogenital distress (*p* < 0.05). In addition, comparison between the stabilization exercises combined with PFMT group and the standard PFMT group revealed a significant difference in pretreatment values (*p* < 0.05) (Table 7).

When differences in ISI scores were examined between groups, significant differences were observed between the stabilization exercises combined with PFMT group and the control group, and between the standard PFMT group and the control group (*p* < 0.05), indicating a reduction in incontinence severity. The stabilization exercises combined with PFMT group and the standard PFMT group showed similar levels of incontinence severity (*p* > 0.05) (Table 7).

When differences in TAI scores were examined between groups, a significant difference was observed between the stabilization exercises combined with PFMT group and the control group (*p* < 0.05), indicating a reduction in anxiety levels. The stabilization exercises combined with PFMT group and the standard PFMT group had similar anxiety levels (*p* > 0.05), as did the standard PFMT group and the control group (*p* > 0.05) (Table 7).

When differences in I-QOL scores were examined between groups, significant differences were observed between the stabilization exercises combined with PFMT group and the control group, and between the standard PFMT group and the control group (*p* < 0.05), indicating an improvement in quality of life. The stabilization exercises combined with PFMT group and the standard PFMT group had similar quality-of-life scores (*p* > 0.05) (Table 7).

When pretreatment and post-treatment UDI scores were examined within the groups, the stabilization exercises combined with PFMT group showed a significant decrease in urogenital distress levels. In contrast, the control group showed a significant increase (*p* < 0.05) (Table 8).

When pretreatment and post-treatment ISI scores were examined within the groups, participants in the stabilization exercises combined with PFMT group showed a significant decrease in incontinence severity. In contrast, the control group showed a significant increase (*p* < 0.05) (Table 8).

When pretreatment and post-treatment TAI scores were examined within the groups, participants in the stabilization exercises combined with PFMT group showed a significant decrease in anxiety levels (*p* < 0.05) (Table 8).

When pretreatment and post-treatment I-QOL scores were examined within the groups, participants in both the stabilization exercises combined with PFMT group and the standard PFMT group showed significant increases in I-QOL scores. In contrast, the control group showed a significant decrease in I-QOL scores (*p* < 0.05) (Table 8).

## 4. Discussion

The present randomized controlled trial investigated the effects of pelvic floor muscle training (PFMT) combined with stabilization exercises on urinary symptoms, quality of life, and anxiety in individuals with chronic low back pain (CLBP) and urinary incontinence. The main finding of this study is that PFMT combined with stabilization exercises resulted in more pronounced improvements in urinary parameters than the standard PFMT group and the no-intervention group, highlighting the potential added value of an integrated lumbopelvic rehabilitation approach.

### 4.1. Urogenital Distress (UDI)

The most prominent improvement observed in this study was a reduction in urogenital distress, particularly in the group receiving stabilization exercises combined with PFMT. This finding suggests that addressing trunk stabilization alongside pelvic floor function may enhance symptom relief beyond pelvic floor training alone. Similar improvements in urinary symptom burden following combined PFMT and stabilization-based interventions have been reported in previous randomized controlled trials involving women with urinary incontinence and CLBP, supporting a synergistic therapeutic effect [12,13]. From a mechanistic perspective, improved trunk motor control and coordinated activation of the deep abdominal and pelvic floor muscles may reduce dysregulated intra-abdominal pressure and enhance urethral support, particularly relevant for individuals with CLBP who often exhibit impaired motor control patterns [13].

### 4.2. Incontinence Severity (ISI)

Incontinence severity decreased significantly in both intervention groups compared with the control group; however, no significant difference was observed between the PFMT combined with stabilization exercises group and the standard PFMT group. This finding is consistent with previous evidence demonstrating that PFMT alone can effectively reduce incontinence severity when adequate contraction intensity and repetition are achieved [32]. Studies examining different training intensities and program durations suggest that longer intervention periods or higher training frequencies may be required to detect between-group differences when PFMT is compared with combined exercise approaches [13,32]. Accordingly, the similar ISI outcomes observed between the two intervention groups in the present study may reflect intervention duration rather than the absence of an added effect of stabilization exercises.

### 4.3. Anxiety (TAI)

Anxiety levels improved significantly only in the group receiving stabilization exercises combined with PFMT when compared with the control group. This finding suggests that integrated exercise programs targeting both postural control and pelvic floor function may have beneficial effects that extend beyond physical symptoms. Previous multidisciplinary and combined-intervention studies have reported greater reductions in anxiety and psychosocial distress when PFMT is supplemented with interventions addressing postural stability or behavioral components. Although no formal psychological intervention was included in the present study, the observed reduction in anxiety may reflect increased body awareness, improved postural control, and enhanced confidence in symptom management resulting from stabilization-based training [33].

### 4.4. Quality of Life (I-QOL)

Quality of life improved significantly in both intervention groups, with no significant difference between the standard PFMT group and the PFMT combined with stabilization exercises. This finding aligns with previous studies reporting that improvements in urinary symptoms are closely associated with enhancements in disease-specific quality-of-life measures following PFMT-based interventions [12,32]. While the combined intervention did not demonstrate superiority over PFMT alone for quality-of-life outcomes, the significant within-group improvements observed in both intervention groups underscore the effectiveness of pelvic floor–focused rehabilitation in improving daily functioning and well-being.

### 4.5. Clinical Implications and Directions for Future Research

The findings of this study support the clinical use of PFMT as a core rehabilitation strategy for individuals with urinary incontinence and CLBP. Importantly, the addition of stabilization exercises appears to provide added benefits for reducing urogenital distress and anxiety, suggesting that clinicians may consider integrated lumbopelvic exercise programs when managing complex clinical presentations involving both spinal and pelvic floor dysfunction [12,13]. Such combined approaches may be particularly valuable in patients presenting with motor control deficits, postural instability, or increased psychosocial burden.

### 4.6. Limitations

Although a baseline difference in UDI scores was observed between intervention groups, additional analyses based on change scores yielded consistent between-group patterns, indicating that the findings were unlikely to be driven by initial group differences.

Several limitations should be considered when interpreting the results of this study. The relatively small sample size and short intervention duration may have limited the detection of between-group differences for some outcomes. Outcome assessments were performed by the same physiotherapist who supervised the intervention and was therefore not blinded to group allocation. Participants were also not blinded due to the nature of the exercise interventions. Although allocation concealment was ensured using sequentially numbered, opaque, sealed envelopes, the absence of assessor blinding may have introduced measurement bias. Future studies incorporating blinded outcome assessment and longer follow-up periods are warranted to further strengthen the evidence base.

## 5. Conclusions

This randomized controlled trial demonstrated that pelvic floor muscle training combined with stabilization exercises resulted in greater improvements in urinary parameters, particularly urogenital distress, compared with pelvic floor muscle training alone and no intervention in individuals with chronic low back pain and urinary incontinence. Both intervention approaches were effective in improving urinary symptoms and quality of life when compared with the control condition.

From a clinical perspective, these findings support the use of pelvic floor muscle training as a fundamental rehabilitation strategy and suggest that incorporating stabilization exercises may provide additional benefits, especially for reducing urogenital distress and anxiety. Integrated stabilization exercise programs may therefore be considered in clinical practice when managing patients with concurrent spinal and pelvic floor dysfunction.

## Figures and Tables

**Table 1:** The pelvic floor muscle training program combined with stabilization exercises.

PFMT Combined with Stabilization Exercises	First and Second Week	Third and Fourth Week	Fifth and Sixth Week	Seventh and Eighth Week
	Week 1: 8–12 Reps × 2 Sets Week 2: 15–20 Reps × 2 Sets	Week 3: 8–12 Reps × 2 Sets Week 4: 15–20 Reps × 2 Sets	Week 5: 8–12 Reps × 3 Sets Week 6: 15–20 Reps × 3 Sets	Week 7: 8–12 Reps × 3 Sets Week 8: 15–20 Reps × 3 Sets
Bridge	Beginner level	Single leg lift (bilateral)	With ball squeeze	With elastic band resistance
Supine abdominal	Beginner level	With arms extended	With arms crossed	Hands behind head
Dead bug exercise	Legs only	Arms and legs	Arms and legs (leg resistance)	Arms and legs (arm and leg resistance)
Bird dog exercise	Arm and leg separate	Cross arm and leg	Cross arm and leg (leg resistance)	Cross arm and leg (arm and leg resistance)
Clamshell	-	Beginner level	With elastic band resistance	With hip lift
Squat	-	-	Mini squat	Deep squat
Plank	-	-	Wide stance	Narrow stance
Side plank	-	-	Knees bent	Knees extended

PFMT, pelvic floor muscle training; Reps, Repetitions.

**Table 2:** Standard pelvic floor muscle training program.

Standard Pelvic Floor Muscle Training	First and Second Week	Third and Fourth Week	Fifth and Sixth Week	Seventh and Eighth Week
	Week 1: 8–12 Reps × 2 Sets Week 2: 15–20 Reps × 2 Sets	Week 3: 8–12 Reps × 2 Sets Week 4: 15–20 Reps × 2 Sets	Week 5: 8–12 Reps × 3 Sets Week 6: 15–20 Reps × 3 Sets	Week 7: 8–12 Reps × 3 Sets Week 8: 15–20 Reps × 3 Sets
Exercise positions	Supine	Side lying	Prone	Quadruped
	Side lying	Prone	Quadruped	Sitting
	Prone	Quadruped	Sitting	Standing

Reps, repetitions.

**Table 3:** Demographic and physical characteristics of the participants.

	Stabilization Exercises Combined with PFMT Group n = 16	Standard PFMT Group n = 13	Control Group n = 15	χ^2^	*p*
Age (years) (X ± SD) (Minimum–maximum)	45.06 ± 7.20 34–59	47.85 ± 6.93 41–65	44.13 ± 8.44 25–57	1.189	0.552
BMI (kg/m^2^) (X ± SD) (Minimum–maximum)	24.96 ± 2.34 20.6–28.6	25.96 ± 1.83 23.5–28.7	24.90 ± 2.87 19.2–29	1.632	0.442
Symptom duration (months) (X ± SD) (Minimum–maximum)	6.88 ± 3.79 3–14	7.15 ± 3.63 3–12	6.80 ± 3.10 3–13	0.005	0.998

PFMT, pelvic floor muscle training; BMI, body mass index; χ^2^, Kruskal–Wallis test statistic; *p* < 0.05.

**Table 4:** Investigation of urinary parameters and quality-of-life measures.

Urinary Symptoms		Stabilization Exercises Combined with PFMT Group n = 16	Standard PFMT Group n = 13	Control Group n = 15	χ^2^	*p*
		(X ± SD)	(X ± SD)	(X ± SD)		
UDI (0–18)	Pretreatment	8.44 ± 2.34	10.77 ± 2.35	10.07 ± 4.35	5.428	0.066
	Post-treatment	6.25 ± 3.28	9.15 ± 2.38	11.33 ± 2.80	15.599	0.001
ISI (1–12)	Pretreatment	6.56 ± 3.85	6.38 ± 3.04	5.27 ± 2.82	1.199	0.549
	Post-treatment	3.00 ± 1.75	4.46 ± 2.60	7.33 ± 2.58	16.987	0.001
TAI (20–80)	Pretreatment	49.87 ± 8.40	46.08 ± 6.58	47.53 ± 7.53	1.296	0.523
	Post-treatment	42.25 ± 3.61	45.54 ± 8.35	49.40 ± 7.12	8.102	0.017
I-QOL (22–110)	Pretreatment	59.06 ± 18.54	61.92 ± 13.23	66.33 ± 14.25	2.062	0.357
	Post-treatment	77.19 ± 13.15	74.15 ± 10.63	55.73 ± 14.43	14.718	0.001

PFMT, pelvic floor muscle training; UDI, Urogenital Distress Inventory; ISI, Incontinence Severity Index; TAI, Trait Anxiety Inventory; I-QOL, Incontinence Quality-of-Life Scale; χ^2^, Kruskal–Wallis test statistic; *p* < 0.05.

**Table 5:** Effect Sizes (r) and 95% Confidence Intervals for post-treatment Between-Group Comparisons.

Urinary Symptoms		Stabilization Exercises Combined with PFMT vs. Standard PFMT			Stabilization Exercises Combined with PFMT vs. Control Comparison			Standard PFMT vs. Control Comparison		
		r (|z|/√N)	95% CI Lower	95% CI Upper	r (|z|/√N)	95% CI Lower	95% CI Upper	r (|z|/√N)	95% CI Lower	95% CI Upper
UDI	Post-treatment	0.46	0.10	0.72	0.65	0.39	0.81	0.37	0.02	0.65
ISI	Post-treatment	0.27	−0.10	0.57	0.73	0.51	0.86	0.47	0.10	0.70
TAI	Post-treatment	0.18	−0.23	0.50	0.51	0.19	0.73	0.29	−0.07	0.58
I-QOL	Post-treatment	0.15	−0.23	0.50	0.63	0.36	0.80	0.56	0.21	0.74

PFMT, pelvic floor muscle training; UDI, Urogenital Distress Inventory; ISI, Incontinence Severity Index; TAI, Trait Anxiety Inventory; I-QOL, Incontinence Quality-of-Life Scale. Effect sizes were calculated as r = |Z|/√N based on the Z statistic derived from the Mann–Whitney U test. 95% CIs (confidence intervals) were computed using Fisher’s z transformation. According to conventional benchmarks, r = 0.10 represents a small effect, r = 0.30 a moderate effect, and r ≥ 0.50 a large effect.

**Table 6:** Between-Group Comparisons of Change Scores (Post–Pre) for Urinary and Quality-of-Life Outcomes.

Outcome	Kruskal–Wallis *p*	Significant Pairwise Comparisons
ΔUDI	0.002	Stab + PFMT > Standard (*p* < 0.05); Stab + PFMT > Control (*p* < 0.01)
ΔISI	<0.001	Stab + PFMT > Standard; Stab + PFMT > Control
ΔTAI	0.042	Stab + PFMT > Standard
ΔI-QOL	<0.001	Stab + PFMT > Standard; Stab + PFMT > Control

Δ indicates change score (post-treatment minus pretreatment). Between-group comparisons were performed using the Kruskal–Wallis test, followed by Mann–Whitney U tests for pairwise comparisons when appropriate.

**Table 7:** Comparison of urinary parameters and quality of life between groups.

Urinary Parameters		Stabilization Exercises Combined with PFMT vs. Standard PFMT		Stabilization Exercises Combined with PFMT vs. Control Comparison		Standard PFMT vs. Control Comparison	
		z	*p*	z	*p*	z	*p*
UDI	Pretreatment	−2.363	0.018	−1.392	0.164	−0.742	0.458
	Post-treatment	−2.448	0.014	−3.611	0.000	−1.995	0.046
ISI	Pretreatment	−0.089	0.929	−0.762	0.446	−1.119	0.263
	Post-treatment	−1.433	0.152	−4.040	0.000	−2.502	0.012
TAI	Pretreatment	−1.119	0.263	−0.713	0.476	−0.392	0.695
	Post-treatment	−0.968	0.333	−2.893	0.004	−1.547	0.122
I-QOL	Pretreatment	−0.790	0.429	−1.286	0.199	−0.899	0.369
	Post-treatment	−0.812	0.417	−3.500	0.000	−2.952	0.003

PFMT, pelvic floor muscle training; UDI, Urogenital Distress Inventory; ISI, Incontinence Severity Index; TAI, Trait Anxiety Inventory; I-QOL, Incontinence Quality of Life Scale; z, Mann–Whitney U Test, *p* < 0.05.

**Table 8:** Within-group comparisons of urinary parameters and quality of life.

Urinary Parameter	Stabilization Exercises Combined with PFMT Group n = 16				Standard PFMT Group n = 13				Control Group n = 15			
	Pre-T (X ± SD)	Post-T (X ± SD)	z	*p*	Pre-T (X ± SD)	Post-T (X ± SD)	z	*p*	Pre-T (X ± SD)	Post-T (X ± SD)	z	*p*
UDI	8.44 ± 2.34	6.25 ± 3.28	−2.481	0.013	10.77 ± 2.35	9.15 ± 2.38	−1.697	0.090	10.07 ± 4.35	11.33 ± 2.80	−1.220	0.222
ISI	6.56 ± 3.85	3.00 ± 1.75	−3.191	0.001	6.38 ± 3.04	4.46 ± 2.60	−1.929	0.054	5.27 ± 2.82	7.33 ± 2.58	−2.365	0.018
TAI	49.87 ± 8.40	42.25 ± 3.61	−3.033	0.002	46.08 ± 6.58	45.54 ± 8.35	−0.210	0.834	47.53 ± 7.53	49.40 ± 7.12	−0.818	0.414
I-QOL	59.06 ± 18.54	77.19 ± 13.15	−3.082	0.002	61.92 ± 13.23	74.15 ± 10.63	−3.112	0.002	66.33 ± 14.25	55.73 ± 14.43	−3.069	0.002

PFMT, pelvic floor muscle training; UDI, Urogenital Distress Inventory; ISI, Incontinence Severity Index; TAI, Trait Anxiety Inventory; I-QOL, Incontinence Quality of Life Scale; z, Wilcoxon signed-rank test, *p* < 0.05.

## Data Availability

The data presented in this study are available from the corresponding author upon reasonable request.

## Supplementary Materials

The following supporting information can be downloaded at: https://www.mdpi.com/article/10.3390/jcm15062333/s1, Figures S1 and S2: Examples of stabilization exercises.

## Abbreviations

The following abbreviations are used in this manuscript:

BMI	Body mass index
CLBP	Chronic Low Back Pain
ISI	Incontinence Severity Index
I-QOL	Incontinence Quality of Life Questionnaire
NRS	Numeric Rating Scale
PFMT	Pelvic floor muscle training
TAI	Trait Anxiety Inventory
UDI	Urogenital Distress Inventory
