# Supplementary figures and images for: Effects of Stabilization Exercises and Pelvic Floor Muscle Training on Urinary Parameters in Individuals with Chronic Low Back Pain and Urinary Incontinence: A Randomized Controlled Trial
Source: J Clin Med. 2026 Mar 18;15(6):2333. doi: 10.3390/jcm15062333 (PMC13027224; doi:10.3390/jcm15062333)

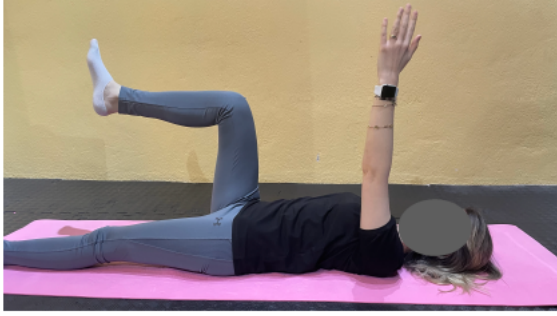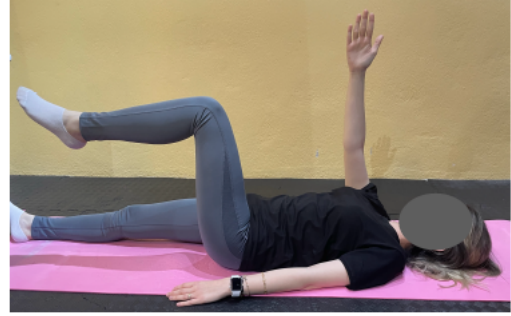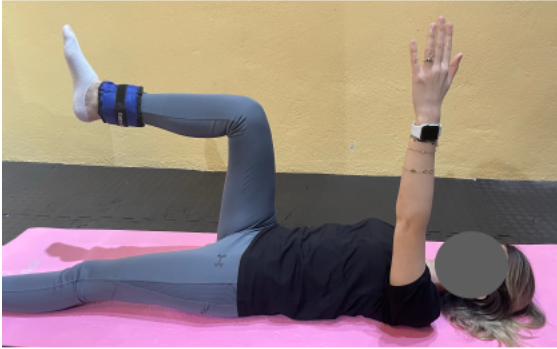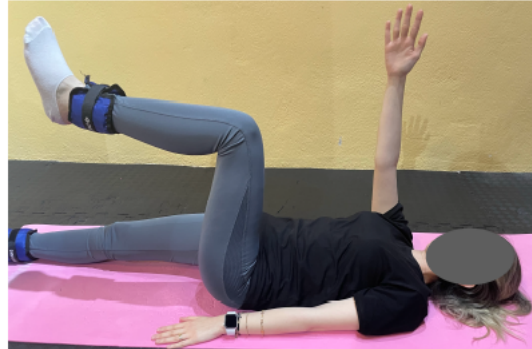

Figure S1

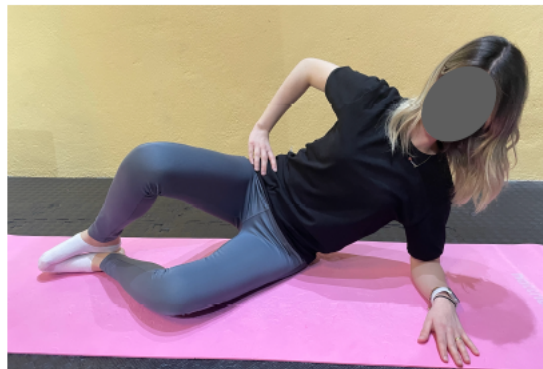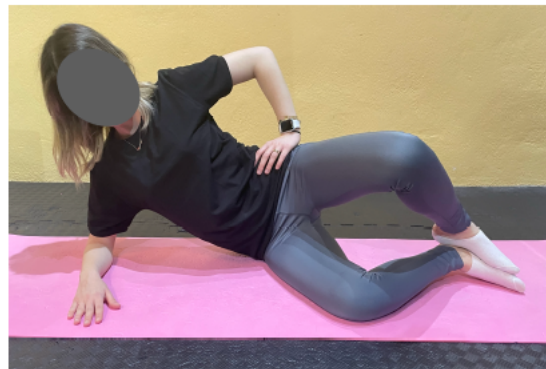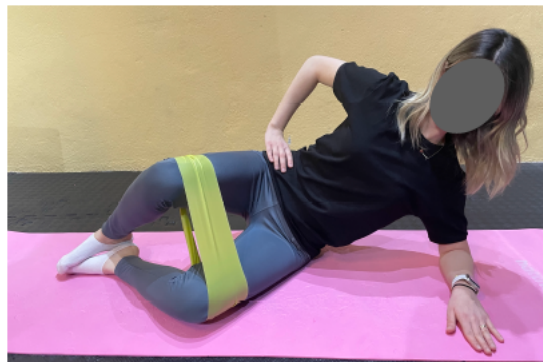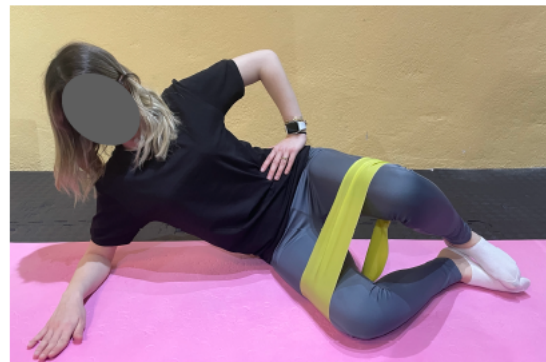

Figure S2

Supplement: Supplementary file 1 [file jcm-15-02333-s001.zip › jcm-4137474-supplementary.pdf]
